# Joint Binding of OTX2 and MYC in Promotor Regions Is Associated with High Gene Expression in Medulloblastoma

**DOI:** 10.1371/journal.pone.0026058

**Published:** 2011-10-10

**Authors:** Jens Bunt, Nancy E. Hasselt, Danny A. Zwijnenburg, Jan Koster, Rogier Versteeg, Marcel Kool

**Affiliations:** Department of Oncogenomics, Academic Medical Center, Amsterdam, The Netherlands; Instituto Nacional de Câncer, Brazil

## Abstract

Both OTX2 and MYC are important oncogenes in medulloblastoma, the most common malignant brain tumor in childhood. Much is known about MYC binding to promoter regions, but OTX2 binding is hardly investigated. We used ChIP-on-chip data to analyze the binding patterns of both transcription factors in D425 medulloblastoma cells. When combining the data for all promoter regions in the genome, OTX2 binding showed a remarkable bi-modal distribution pattern with peaks around −250 bp upstream and +650 bp downstream of the transcription start sites (TSSs). Indeed, 40.2% of all OTX2-bound TSSs had more than one significant OTX2-binding peak. This OTX2-binding pattern was very different from the TSS-centered single peak binding pattern observed for MYC and other known transcription factors. However, in individual promoter regions, OTX2 and MYC have a strong tendency to bind in proximity of each other. OTX2-binding sequences are depleted near TSSs in the genome, providing an explanation for the observed bi-modal distribution of OTX2 binding. This contrasts to the enrichment of E-box sequences at TSSs. Both OTX2 and MYC binding independently correlated with higher gene expression. Interestingly, genes of promoter regions with multiple OTX2 binding as well as MYC binding showed the highest expression levels in D425 cells and in primary medulloblastomas. Genes within this class of promoter regions were enriched for medulloblastoma and stem cell specific genes. Our data suggest an important functional interaction between OTX2 and MYC in regulating gene expression in medulloblastoma.

## Introduction

*OTX2* encodes a homeodomain containing transcription factor, which is essential for normal brain development. In the cerebellum, *OTX2* is expressed in progenitor cells, but only at pre-natal stages. In post-natal cerebellum, no OTX2 expression can be detected [1]. However, the malignant childhood brain tumor medulloblastoma, which originates in the cerebellum, often expresses OTX2 at high levels [1]–[5]. These high OTX2 levels together with amplification or gain of the *OTX2* locus in a subset of the tumors suggest an oncogenic role for OTX2 in medulloblastoma [3], [4], [6], [7].

Indeed, we and others have shown a dependency on OTX2 for medulloblastoma cells with *OTX2* expression [7], [8]. Cells in which we silenced OTX2 expression were inhibited in proliferation and started to differentiate [8]. Expression profiling after induction of ectopic OTX2 or silencing endogenous OTX2 in medulloblastoma cell lines revealed over 2000 genes regulated downstream of OTX2, including many cell cycle and eye developmental genes [8], [9]. ChIP-on-chip analyses identified cell cycle genes as major direct targets of OTX2, while differentiation genes, strongly regulated after OTX2 silencing, appeared to be indirect targets.

Although OTX2 is an essential gene in medulloblastoma, little is known about the mechanism by which OTX2 regulates the expression of its target genes. Only in a few studies interaction of OTX2 with other gene expression regulators have been reported. These studies mainly focused on the regulation of a single target gene [10]–[15]. Early studies used limited promoter regions or oligonucleotides to assess OTX2 binding and they identified TAATCC and related sequences as the main DNA-binding motif for OTX2 [16]–[21].

In this study, we have analyzed the binding of OTX2 to promoter regions in the complete genome and compared the OTX2 data with ChIP-on-chip data for MYC in medulloblastoma. MYC is another important oncogene in medulloblastoma pathogenesis and high-level amplifications of the MYC locus are significantly associated with a poor clinical outcome [22]–[25]. Our analyses show that 40.2% of all OTX2 bound promoter regions contain multiple OTX2-binding peaks, while the other 59.8% showed only single OTX2 binding. Together they contribute to a specific bi-modal distribution of OTX2 binding near TSSs. The distribution of OTX2-binding motifs provided a possible explanation for the bi-modal distribution as these motifs were depleted near TSSs in the genome. MYC binding displayed a distribution centered on the TSS, similar to other studies [26], [27]. Both OTX2 and MYC binding to the promoter region correlated with higher gene expression. The highest expression levels were found for genes with multiple OTX2-binding peaks plus MYC binding. These genes, but not the genes with single OTX2 binding or the genes with multiple OTX2 binding and no MYC, were enriched for medulloblastoma and stem cell specific genes. Furthermore, OTX2 and MYC bind closer to each other in promoter regions than would have been expected if binding occurred randomly. Together these results suggest that OTX2 and MYC cooperate in regulating gene expression in medulloblastoma.

## Results

### OTX2 shows unique DNA-binding pattern in medulloblastoma cell lines

**Figure 1:**
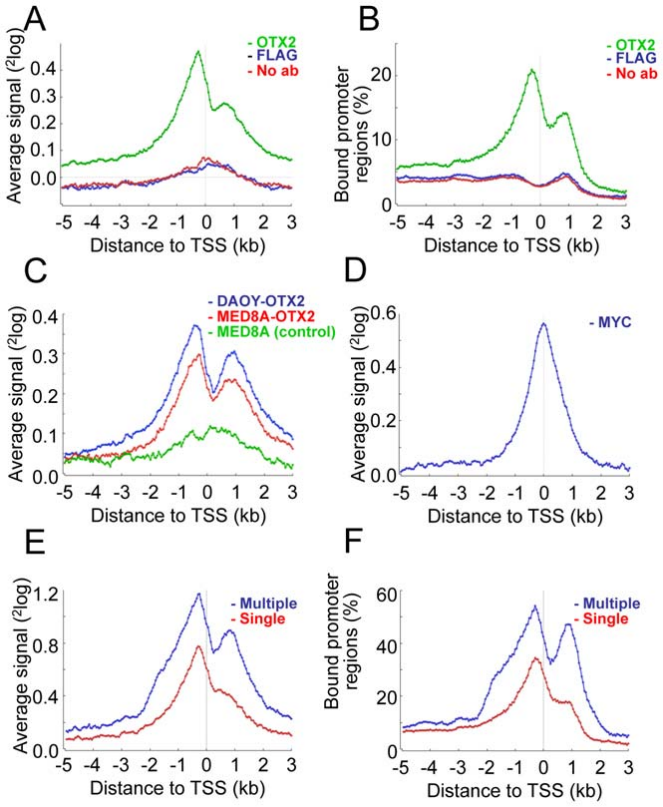
OTX2 binding reveals a bi-modal distribution surrounding transcription start sites. A. Graphical representation of the average ^2^log signal of OTX2 ChIP-on-chip data in D425 medulloblastoma cells (green line) as a function of the distance to the transcription start site (TSS). OTX2 binding displays a bi-modal distribution, which is absent in the controls using a FLAG or no antibody (blue and red line, respectively). B. The percentage of promoter regions with OTX2-binding peaks were plotted as a function of the distance to the TSS. This also shows a bi-modal distribution of OTX2. C. A bi-modal distribution of OTX2 binding was also found using OTX2 ChIP-on-chip data from MED8A and DAOY medulloblastoma cell lines with ectopic OTX2 overexpression (red and blue lines, respectively). MED8A cells without OTX2 (green line) did not show this pattern. D. Average MYC binding in D425 cells peaks at the TSS. E. Average OTX2-binding signal plotted separately for promoter regions with single (red line) or multiple OTX2-binding peaks (blue line). F. Percentage of bound promoters as a function of the distance from the TSS for promoters with single OTX2-binding peaks (red line) and promoters with multiple OTX2-binding peaks (blue line).

To investigate OTX2 binding, we examined where OTX2 binds in promoter regions. Data from our OTX2 ChIP-on-chip (Nimblegen promoter arrays) experiment in D425 cells were used [8]. To get a global overview of OTX2 binding we aligned all promoter regions according to the position of the transcriptional start sites (TSSs) and per bin of 50 bp we calculated the average OTX2-binding signal for all promoters together. The results revealed a bi-modal distribution of OTX2 binding in promoter regions with peaks around −250 bp upstream and +650 bp downstream from the TSSs (Figure 1A).

A similar bi-modal distribution was found when only considering the significant binding peaks (as called by the Nimblegen algorithm). We plotted these data as the percentage of all promoter regions with a significant binding peak and observed a similar bi-modal distribution (Figure 1B). The two control ChIP-on-chip experiments, in which we omitted the OTX2 antibody or used a FLAG antibody instead, did not show this pattern. A similar bi-modal distribution of OTX2 binding was also observed when we used ChIP-on-chip data of MED8A and DAOY medulloblastoma cells with ectopic OTX2 expression (Figure 1C and Figure S1) [9].

As this OTX2 binding distribution strongly differs from the TSS-centered single peak pattern commonly found for transcription factors, we performed another ChiP-on-chip experiment for MYC, which is like OTX2 also amplified and highly expressed in D425 cells (Figure S1). Data from this ChIP-on-chip experiment showed that the MYC binding, calculated over all promoter regions, is concentrated in a single peak centered on the TSS (Figure 1D), in line with previous reports for MYC in other cell systems [26], [27].

To investigate whether the bi-modal distribution of OTX2 implies multiple OTX2 binding peaks per gene, we calculated the number of significant OTX2-binding peaks per promoter region. As a result, 40.2% of the 11,389 promoter regions with OTX2 binding had two or more significant OTX2-binding peaks, and the remaining 59.8% had a single OTX2-binding peak. For both single and multiple OTX2-bound promoter regions the widths of the peaks were similar. The promoter regions with multiple OTX2 binding displayed again the bi-modal distribution pattern (Figure 1E and 1F). Even when correcting for overlapping promoter regions, a bi-modal pattern remained. In single bound promoter regions, there was a less prominent bi-modal distribution (Figure 1E and 1F). However, the distribution of single OTX2-bound promoters did not resemble the MYC-binding pattern, which is TSS-centered. Therefore, the overall bi-modal distribution seems not caused by multiple OTX2 binding alone, but a general tendency of OTX2 to bind adjacent to the TSS.

### OTX2 and MYC bind close to each other in promoter regions

As both OTX2 and MYC bind to many genes in the genome [8], [26]–[28], we examined to what extent OTX2 and MYC bind the same promoter regions. Out of 25,064 promoter regions present on the Nimblegen chip, 11,389 (45.4%) had one (6,811) or more (4,578) OTX2-binding peaks within 2 kb of the TSS. MYC-binding was detected for 12,906 (51.5%) promoter regions and 6,240 (24.9%) had both OTX2 and MYC binding. The most significant overlap was found for promoter regions with multiple OTX2-binding peaks. Of these, 63.8% (2,923 of 4,578) also had MYC binding (p = 6.9*E-78 hypergeometric test compared to all promoter regions). For promoter regions with a single OTX2-binding peak, we detected MYC binding in 48.7% (3,317 of 6,811) of the cases. Hence, based on the amount of OTX2-binding peak and MYC binding, four different classes of OTX2-bound promoters were defined: single OTX2-bound promoter regions with or without MYC binding and multiple OTX2-bound promoter regions with or without MYC binding.

**Figure 2:**
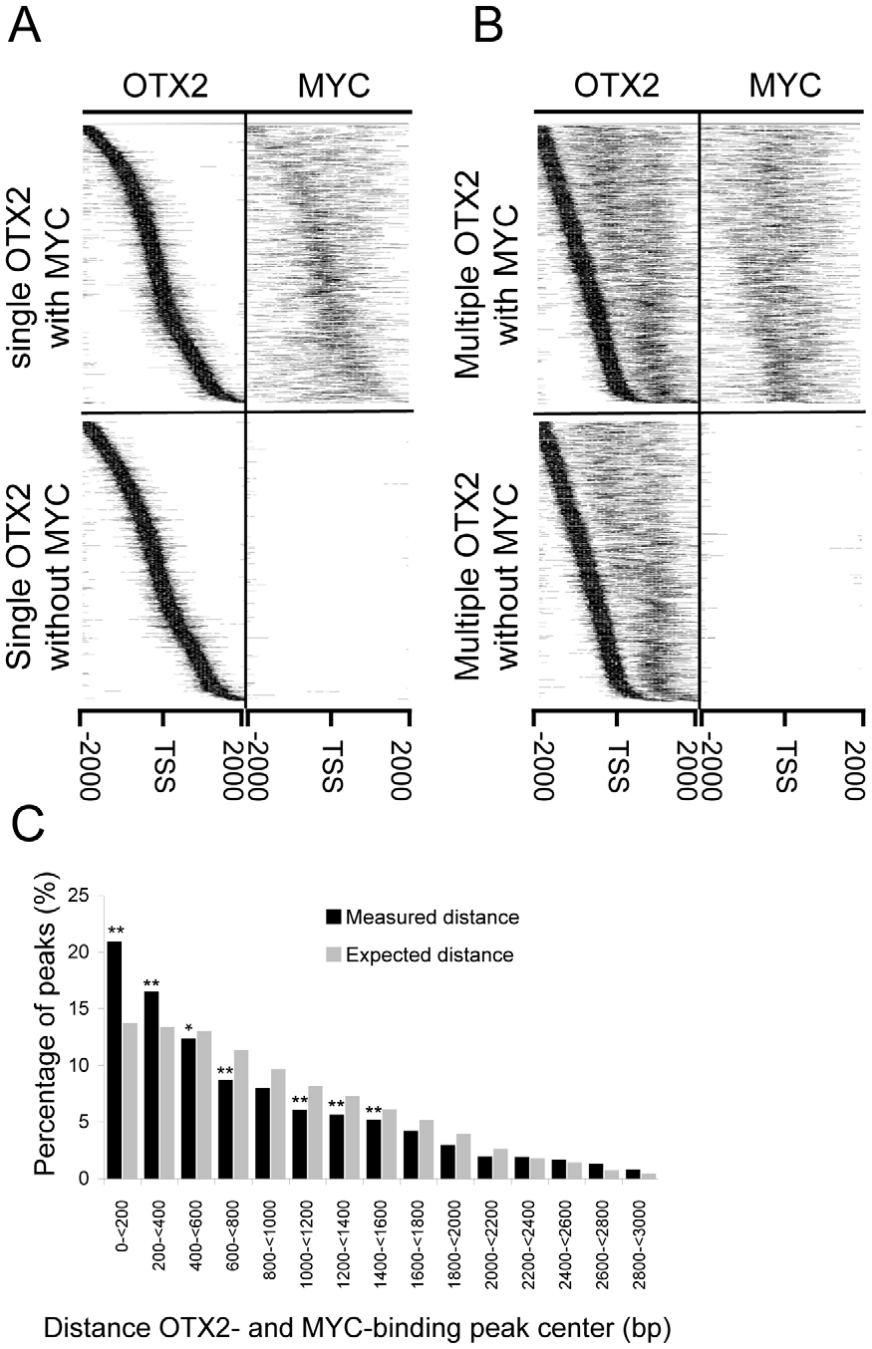
OTX2 and MYC bind close to each other in promoter regions. For each of the four classes of promoter regions, the ^2^log binding signals of OTX2- and MYC-binding peaks are shown. Promoter regions are sorted by the location of the first upstream OTX2-binding peak. A. Both classes with a single OTX2-binding peak have a very similar OTX2-binding pattern. When MYC is also bound the MYC-binding peaks closely follow the OTX2 pattern. B. Promoter regions with multiple OTX2-binding peaks. MYC-binding peaks again accumulate there where OTX2 is bound. C. The distances between OTX2- and MYC-binding peak centers were calculated for all promoter regions with single OTX2 binding with MYC binding. The percentage of OTX2-binding peaks within different distance ranges was plotted. Data show that significantly more peaks are close to each other (within 400 bp from each other) then expected (* <0.05, ** <0.0005, Fisher's exact test). Expected distance was generated by multiple randomization of the OTX2 position.

Figure 2 shows the OTX2-binding peaks for all four possible combinations of OTX2 and MYC binding to promoter regions with OTX2 binding. Two classes have single OTX2-binding peaks with or without MYC binding (Figure 2A), while the other two classes have multiple OTX2 peaks with or without MYC binding (Figure 2B). Per class the promoter regions were sorted according to the location of the first OTX2 binding peak, starting with the most upstream peak. Strikingly, when we depicted the MYC-binding peaks for these same promoter regions (Figure 2A and 2B, right panels), we observed that the MYC-binding peaks closely follow the OTX2-binding pattern. This was most clear for the class of promoter regions with single OTX2-binding peaks, but even for the class of promoter regions with multiple OTX2 binding the MYC-binding peaks tend to accumulate in the same areas. Reversely, when viewing from the MYC binding perspective, OTX2 binding follows MYC binding particularly when closer to the TSS (Figure S2). These data suggest a frequent co-localization of both transcription factors on the promoter. Furthermore, when comparing the calculated distance between OTX2 and MYC-binding peaks to the expected distance (Figure 2C) OTX2 and MYC bind closer to each other in promoter regions then expected. However, they do not occupy the exact same location. MYC preferably binds near or at the TSS, while OTX2 binding tends to be more adjacent to the TSS.

### OTX2 binding motifs are depleted near the TSSs

**Figure 3:**
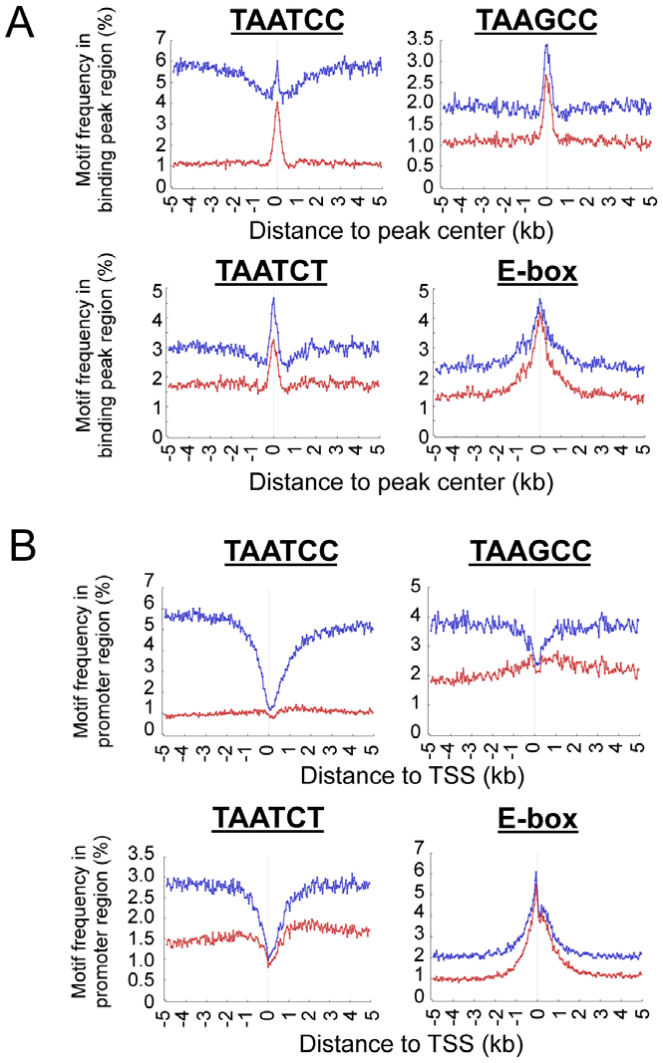
OTX2 motifs enriched at OTX2 peak centers, but not at TSSs. A. The percentage of the OTX2-binding motifs (TAATCC, TAAGCC and TAATCT) per 50 bp were plotted in regards to the OTX2-binding peak center. For all three motifs, enrichment at the center of OTX2-binding peaks is observed, although for TAATCC this was more apparent in repeat masked data (red) compared to unmasked data (blue). Similar results were obtained for the E-box sequences in the MYC experiment. B. The presence of OTX2- and MYC-binding motifs was scored per 50 bp for all promoter regions in the genome. While E-box sequences display a higher frequency near TSSs in the genome, TAATCC, TAAGCC and TAATCT do not. They all show a clear depletion near TSSs, which is most clear in data without repeat mask (blue lines) as compared to data with repeat mask (red lines).

We investigated whether the observed bi-modal distribution of OTX2 binding relates to DNA-binding motifs. Therefore, we performed Discriminating Motif Enumerator (DME) analyses of the regions bound by OTX2 to discover binding motifs over-represented within the OTX2-binding peaks [29]. TAATCC turned out to be the most over-represented sequence, followed by the related TAAGCC and TAATCT sequences (Table S1A). The four different promoter classes all showed a similar frequency for these sequences. These same sequences were also enriched in the OTX2-bound sequences identified in MED8A cells with overexpression of ectopic OTX2 (Table S1B). Similar analyses identified known E-box sequences as the main binding motifs in MYC-bound regions (Table S1C). All OTX2 and MYC motifs were enriched in the centers of binding peaks (Figure 3A). We also analyzed the distribution of these OTX2- and MYC-binding motifs in promoter regions. Surprisingly, all three OTX2 motifs were depleted near the TSSs, while E-box sequences were enriched (Figure 3B, blue lines). As TAATCC and related sequences are enriched in Alu repeats, these analyses were repeated with a repeat mask. However, OTX2 motifs still did not peak at the TSSs, while E-box sequences remained enriched (Figure 3B, red lines). Thus, the bi-modal distribution of OTX2 binding might be caused by the depletion of OTX2-binding motifs near the TSSs.

### Binding of OTX2 and MYC is associated with increased gene expression

**Figure 4:**
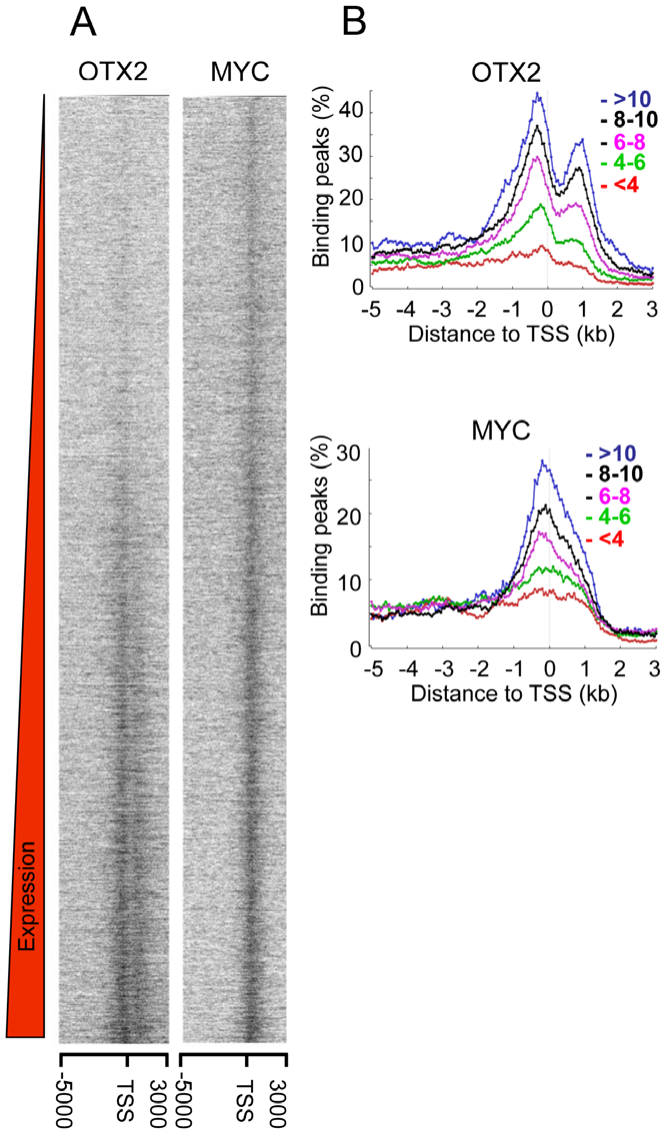
Both OTX2 and MYC binding correlate with high gene expression. A. All individual promoter regions were ordered by expression level of the associated gene in D425 cells and the corresponding ^2^log binding signals of OTX2 and MYC in D425 cells were visualized. Both OTX2 and MYC binding positively correlates with gene expression. B. All promoter regions were categorized into five categories by ^2^log expression values of the corresponding genes. With increasing expression the percentage of bound promoter regions also increases both for OTX2 and MYC. Overall distribution patterns do not change.

To assess whether OTX2 or MYC binding is related to gene expression, we depicted the OTX2- and MYC-binding peaks for each promoter region and sorted the promoter regions according to the gene expression levels as established by Affymetrix expression profiling of D425 cells [8]. Figure 4A (left panel) shows a correlation between the amount of OTX2-binding peaks and gene expression levels. The promoters of highly expressed genes showed more signal. The panel on the right in Figure 4A shows similar data for MYC. Like OTX2, MYC binding correlates with gene expression levels. This is also demonstrated in Figure 4B, in which the percentage of all promoter regions that have a OTX2- or MYC- binding peak at the indicated position are plotted. Promoter regions were divided into five categories based on increasing gene expression levels in D425 cells. For both OTX2 and MYC, the percentage of promoter regions with a binding peak increased with increasing expression levels. The bi-modal OTX2 and the TSS-centered MYC- binding patterns do not differ between the different expression categories. Thus, both OTX2 and MYC binding are associated with increased gene expression.

### OTX2 binding motifs do not correlate with expression levels

**Figure 5:**
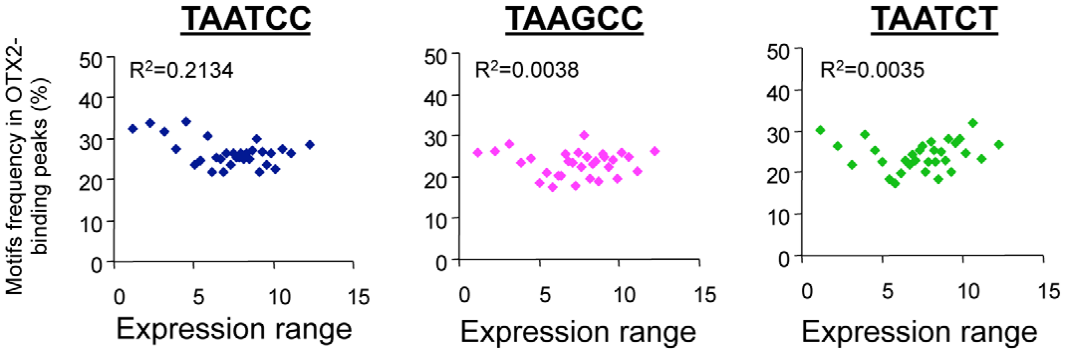
OTX2-binding motifs do not correlate with gene expression levels. All OTX2-binding peaks were sorted by the expression in D425 cells of the associated genes and binned per 400. The motif frequency for each bin is displayed as a function of average expression. For all three OTX2-binding motifs, there is no relation between gene expression levels and motif occurrence within the peaks.

As OTX2 binding correlates with gene expression, we wondered whether the usage of the different OTX2-binding motifs influenced gene expression levels. However, the more highly expressed genes showed no enrichment for any OTX2-binding motifs in their promoter regions (Figure 5). The presence of a motif also had no effect on the number of genes regulated or the magnitude of the regulation after OTX2 silencing in D425 cells (data not shown). Therefore, even though these motifs are required for OTX2 binding, additional factors seem to be necessary to determine expression levels and regulation.

### Genes with multiple OTX2 and MYC binding are expressed at higher levels in medulloblastoma and stem cells

**Figure 6:**
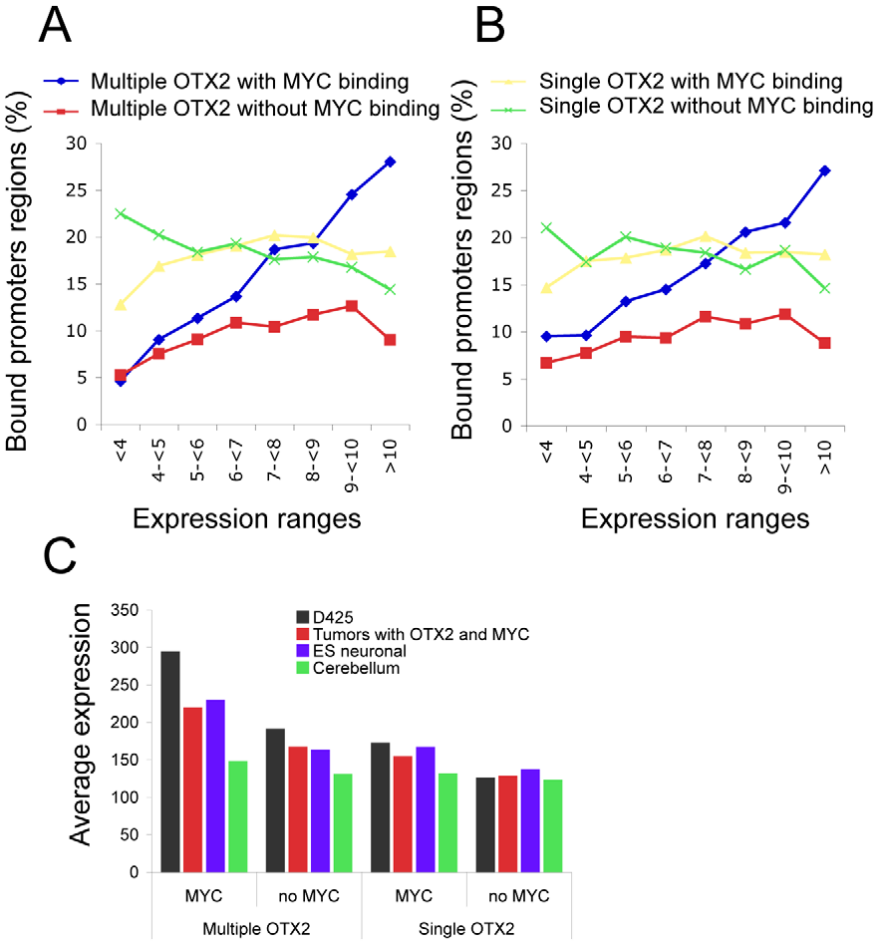
Multiple OTX2-binding peaks with MYC binding is associated with high gene expression. A and B. OTX2-bound promoter regions are classified by single or multiple OTX2-binding peaks with or without additional MYC-binding. For each of these promoter classes the percentage of bound promoter regions was calculated in different expression categories using either expression data from D425 cells (A) or expression data from 10 primary medulloblastoma tumors that have both OTX2 and MYC expression (B). Promoter regions with multiple OTX2-binding peaks and MYC binding were clearly enriched among the gene categories that show higher gene expression both in D425 cells and in tumors. C. Genes within the promoter class with multiple OTX2-binding peaks and MYC binding showed significantly higher expression levels in D425 cells, primary medulloblastoma tumors and human neural embryonic stem cells as compared to genes in other classes (minimal p<1.00E-7, T-test). This difference was not observed using expression data of normal cerebellum.

We then investigated whether the four different classes of promoter regions with OTX2 and MYC binding as depicted in Figure 2 show differences in gene expression levels. Interestingly, the most striking correlation was found for the class of promoter regions with multiple OTX2-binding peaks with MYC binding (Figure 6A; blue line). In D425 cells, with increasing expression the percentage of promoter regions that have multiple OTX2 and MYC binding also increased. Therefore, genes with a promoter region from this class were much more abundant in the high expression categories. Genes with promoter regions from the other classes, *i.e.* with single OTX2-binding peaks with or without MYC binding or with multiple OTX2-binding peaks without MYC binding, were more or less equally distributed over all expression categories (Figure 6A). We found the same correlation between OTX2/MYC binding and gene expression when we used expression data of medulloblastoma tumors [5], [30] (Figure 6B). These results suggest that multiple OTX2 and MYC binding have a synergistic effect on gene expression.

Surprisingly, however, there was no relation between the four classes of promoter regions and gene regulation after OTX2 silencing in D425 cells [8]. The percentage of regulated genes (ranging from 15 to 18%) was more or less equal between the four classes. Furthermore, Gene ontology analyses did not reveal any differences in molecular function between the genes in these four classes. In contrast, gene set enrichment analyses (GSEA), using either DAVID [31] or the BROAD tools [32], [33], revealed that genes with multiple OTX2-binding peaks and MYC binding were significantly enriched for genes associated with mouse (neuronal) stem cells [34] and human medulloblastoma [35] (Table S2). The other groups did not show such enrichments.

Finally, we investigated the relation between this class of promoter regions and medulloblastoma/stem cell specific expression. The average expression levels of genes from the class with both multiple OTX2-binding peaks and MYC binding were consistently higher in medulloblastoma and human neuronal stem cells as compared to genes from other classes (Figure 6C). This difference was not present in normal cerebellum. These data suggest that genes with multiple OTX2-binding peaks and MYC binding in their promoter region may have specific functions in medulloblastoma and/or stem cells.

## Discussion

The specific bi-modal distribution of OTX2 binding around the TSSs, as we investigated in medulloblastoma, strongly differs from the binding patterns of MYC and other transcription factors like GATA1 and TCF4 [26], [27], [36]–[38]. This bi-modal OTX2 pattern was observed both in cells with endogenous OTX2 expression (D425) as well as in cells with induced transgene expression (MED8A and DAOY).

The bi-modal distribution consists both of promoter regions with single and multiple OTX2-binding peaks. The class of promoter regions with both multiple OTX2-binding peaks with MYC binding differed from the other classes, as they were associated with higher gene expression in D425 medulloblastoma cells and in primary medulloblastoma tumors. Moreover, genes within this class of promoter regions were enriched for stem cell and medulloblastoma specific gene expression. Thus, OTX2 seems to have a functional interaction with MYC, which might explain why both genes are frequently co-expressed at high levels in medulloblastoma [5], [6], [22].

Even though the binding pattern might be specific for OTX2, other binding properties were consistent with those found for MYC in D425 cells or from what has been described for other transcription factors [26], [27], [36]–[38]. Similar to genes as MYC or OCT4, OTX2 binds to a large number of genes [26], [39]. This frequent binding does not always result in gene regulation, as usually only for around a third or less of the bound genes the expression levels change after overexpressing or silencing the transcription factor [40]. We obtained similar results in OTX2 silencing and ectopic overexpression experiments [8], [9].

The relation between transcription factors binding to promoter regions and gene expression can be biased by the methods used to determine both binding and regulation. First of all, to assign bound regions to the nearest known genes might be inadequate. It disregards many other forms of gene regulation by, for instance, enhancers, which can be located far away from a gene [40]. Secondly, silencing of a transcription factor is commonly used to identify its target genes. The transcription factor levels are greatly reduced by silencing, but never completely absent. Low levels might be sufficient to retain expression. Together this could hinder adequate interpretation of the effect of OTX2 binding on gene regulation.

Furthermore, binding might not result in regulation, because multiple transcription factors are required in order to regulate gene expression. For instance, in mouse embryonal stem cells, transcription factors as Oct4, Sox2 and Nanog all bind up to 10,000 genes each. However, only a small number of genes bound by all three factors showed evidence of direct transcriptional activation by these transcription factors [39]. A similar mechanism may be envisioned for OTX2. An obvious candidate to interact with OTX2 to activate gene expression is MYC, even though it is not expressed in all OTX2-expressing tumors. Genes with combined multiple OTX2-binding peaks and MYC binding have the highest expression in medulloblastoma. However, these genes were not significantly more regulated after OTX2 silencing, suggestion other transcription factors are also involved.

Direct physical interaction between OTX2 and MYC could not be established (data not shown). Other candidates for cooperative binding with OTX2 are scarce as little is known about protein complexes associated with OTX2. Physical interactions with other transcription factors, like TLE4 and MEIS2, have been reported [10], [12]. Both these genes are expressed in medulloblastoma tumors and cell lines. Interestingly, a MEIS2 like motif, TGACAG, was among the enriched motifs within genes down-regulated after OTX2 silencing. Thus, MEIS2 might be necessary to specify the binding and/or biological function of OTX2 in medulloblastoma. Other proteins with reported physical interaction with OTX2 are less likely candidates. For instance MITF, SOX2, LHX1 and FOXA2, show only limited expression in tumors and are absent in D425 cells [11], [14], [15]. However, structural or functional related genes might substitute for them in medulloblastoma. The GSEA analyses for genes with multiple OTX2-binding peaks and MYC binding in their promoter region also showed enrichment for the SP1, NFY and MAZ binding motifs (Table S2). As these genes are highly expressed in medulloblastoma, they may also interact with OTX2 to regulate gene expression.

The role of DNA-binding sequences in the function of transcription factors remains unclear. For OTX2, TAATCC, TAAGCC and TAATCT are the most enriched binding motifs in medulloblastoma, in accordance with literature [16]–[21]. However, like for other transcription factors, the prevalence of these binding motifs in the genome exceeds the number of detected binding. Also the distribution near the TSSs is not predictive for OTX2-binding pattern, as these sequences are even more prevalent distal of a TSS. Still, it does provide in part an explanation for absence of TSS-centered OTX2-binding peaks.

It remains to be determined what guides OTX2 binding to a motif in the genome. One possible explanation is that not all binding sites are accessible. Epigenetic markers, such as DNA methylation, or packed DNA structure might reduce the affinity or availability of a motif for binding. This could explain the absence of OTX2 binding in Alu repeats in promoter regions. Furthermore, competition of different transcription factors to bind in the same genomic region may explain why not all motifs show OTX2 binding. For example, ETS1 and E2F compete in *MYC* promoter binding. Only after mutation of the E2F site, ETS1 binding could be detected [41]. Alternatively, OTX2 binding could be very transient and may require additional factors to stably bind to DNA. Epigenetic markers associated with activated genes expression or the presence of other components of the transcription complex might be necessary to facilitate its binding.

The binding characteristics of OTX2 raise the question, whether OTX2 only has classical transcription factor function. For other highly expressed transcription factors with frequent binding, like MYC, additional epigenetic functions have been described. Peng *et al.* suggested that OTX2, like CRX, might recruit HAT-containing co-activators such as CBP, P300, and GCN5 via direct interaction with ATXN7 to promote histone acetylation [42], [43]. Unfortunately direct evidence was not shown. However, for the homologue ATXN1 interaction was shown in a yeast2hybrid experiment [44]. *ATXN7* is highly expressed in medulloblastoma tumors and cell lines. OTX2 may function in a similar way in promoting epigenetic changes. With this concept in mind, the similarities between the bi-modal distribution of OTX2 and those of histone modifications such as H3K4me3 and H3K9ac, both marks for active gene expression, could hint to a direct role of OTX2 in histone modification [45], [46].

## Materials and Methods

### ChIP-on-chip

D425 medulloblastoma cells were cultured in MEM medium (Invitrogen, Carlsbad, CA), supplemented with 10% fetal bovine serum, 0.1 mM MEM non essential amino acids, 200 mM glutamine, 100 U/mL penicillin and 100 µg/mL streptomycin (Invitrogen) at 37°C in a humified atmosphere containing 5% CO_2_. Two days after plating, cells were cross-linked with 1% formaldehyde for 10′. After washing, cells were incubated for 5′ in swelling buffer (5 mM PIPES, 85 mM KCl, 0.5% NP40) and passed through a 23G needle. Isolated nuclei were lysed for 10′ in 1 M Tris-HCl/1% SDS/0.5 M EDTA pH 8 on ice. Lysates were sonicated on ice 7×25″ at 30 mA. 3 mL sample was diluted 1∶10 in 1% TritonX-100/150 mM NaCl/50 mM Tris-HCl/2 mM EDTA and cleared for 30′ with 40 µL protein A agarose (Roche) and 125 µL 10 mg/mL haring sperm DNA (Roche Applied Sciences, Basel, Switzerland). 30 µL MYC antibody (Abcam, Cambridge, MA) with 40 µL beads were added to cleared samples and thumbled overnight in cold room. The next day beads were sequentially washed with 0.1% SDS/1% TritonX-100/150 mM NaCl/20 mM Tris-HCl/2 mM EDTA, with the same solution with 500 mM NaCl, with 1% Deoxycholate, 1% NP40/250 mM LiCl/10 mM Tris-HCl/2 mM EDTA and finally with 10 mM Tris-HCl/10 mM EDTA. DNA was eluted in 500 µL 100 mM NaHCO_3_/1% SDS. 20 µL 5 M NaCl was added before decrosslinking at 65°C for 4 h. Next 10 µL 0.5 M EDTA, 20 µL 1 M Tris-HCl pH 6.5 and 2 µL 10 mg/mL Prot K (Roche) were added and incubated at 45°C for 1 h to degrade protein. RNA was degraded by adding 5 µL 10 mg/mL Rnase A (Roche) and incubating for 30′ at 37°C. DNA was purified using Qiagen PCR purification kit (Qiagen, Germantown DE) and quantified with Quant-IT Picogreen (Invitrogen).

The recovered DNA was amplified for labeling as described previously [28]. Labeling of the material, hybridization to the 2.1 M Deluxe Promoter Array, scanning of the arrays and peak calling were performed by Nimblegen, Inc. The ChIP-on-chip experiments with OTX2 in D425, MED8A and DAOY cells were previously published [8], [9].

### Data analyses

All data were scaled as ^2^log signal ratios by the Nimblegen algorithm (Nimblegen) and mapped to transcription start sites (TSSs) in the genome. If a gene has multiple promoter regions, TSSs with less than 150 bp spacing from the previous were discarded. All remaining promoter regions were aligned and the average ^2^log binding signal was calculated over 50 bp bins relative to the TSS. OTX2-binding peaks were called significant by the manufacturer's algorithm. Graphs of the average ^2^log signals and the percentage of peaks as a function of the distance to the TSS were generated in R2 (http://r2.amc.nl). Heatmaps of signals for an individual promoter region were generated in TMEV [47]. Only data of promoter regions with more than 100 bins with informative data within −5000 bp to 3000 bp were included. The promoter regions were sorted based on the expression of the corresponding gene in D425 cells (GSE22875) [8].

Promoter regions harboring at least one binding peak with its center overlapping the region of −2000 to 2000 bp surrounding the TSS, were defined as bound by OTX2 or MYC. Distance between OTX2- and MYC-binding peak centers were calculated for all single OTX2-bound promoter regions. The expected distance between OTX2- and MYC-binding peak centers was generated by randomizing all OTX2-binding peak locations in regards to the TSS locations in Excel and taking the average over 3 randomizations. The percentage of OTX2-binding peaks within different distance ranges was plotted for the calculated and expected values. A Fisher's exact test was used for determining significant changes.

All promoter regions were classified based on the combination of OTX2 and MYC binding within the −2000 bp and 2000 bp region. The average expression of genes within these classes was calculated using data of D425 cells (GSE22875), 10 primary non-WNT/non-SHH medulloblastoma with both high OTX2 and MYC expression (GSE10327 and GSE12992), 9 normal cerebellum samples (GSE3526) and undifferentiated human embryonal cells (GSE9921). Gene set enrichment analyses were performed using the DAVID tool (http://david.abcc.ncifcrf.gov/) and BROAD tool (http://www.broadinstitute.org/gsea) [31]–[33].

### Binding motif discovery and genome distribution

The program DME (v2.0) was employed using the hybrid mode to identify enriched DNA-sequence motifs of length 6, 7 or 8 in the peaks, when compared to a background of peak-flanking sequences [29]. The sequences used to build the best scoring motif were masked in the peak sequences and DME was run again using the same settings. This procedure was repeated 15 times.

The main DNA-binding sequences for OTX2 (TAATCC, TAAGCC and TAATCT) and the E-box sequences were aligned to the UCSC hg18. Their frequencies were plotted in relation to their distance to either a OTX2- and MYC-binding peak center or a TSS. To identify relations between the presence of a DNA-binding sequence and gene expression, all peaks were sorted by the expression level of the associated genes. After binning per 400 peaks, the motif frequencies and the average expression levels were calculated per bin and plotted.

### Supplementary M&M

Western blotting and antibodies used were as previously described [9].

## Supporting Information

Figure S1 **Immunoprecipitation of OTX2 and MYC in medulloblastoma cells.** Western blot analyses of immunoprecipitation of OTX2 and MYC in D425 medulloblastoma cells as well as OTX2 in MED8A and DAOY cells with induced OTX2 expression.
(TIFF)

Figure S2 **OTX2-binding distribution in relation to MYC binding.** All promoter regions of the class with single OTX2-binding peak and MYC binding were sorted by the location of the first upstream MYC-binding peak. To assess the relation between MYC and OTX2 binding, the promoter regions were binned per 400 promoter regions, except for the last bin (517). Per bin, the average OTX2- and MYC-binding signals were calculated in regards to the TSS, normalized to 1 and depicted as a panel. The OTX2-binding signal coincides with the MYC, when the MYC binding signal becomes more proximal to the TSS.
(TIFF)

Table S1 **Enrichment of binding motifs in ChIP experiments.**
(XLS)

Table S2 **Gene enrichment analyses.**
(XLS)
