# Supplementary figures and images for: Joint Binding of OTX2 and MYC in Promotor Regions Is Associated with High Gene Expression in Medulloblastoma
Source: PLoS One. 2011 Oct 10;6(10):e26058. doi: 10.1371/journal.pone.0026058 (PMC3189962; doi:10.1371/journal.pone.0026058)

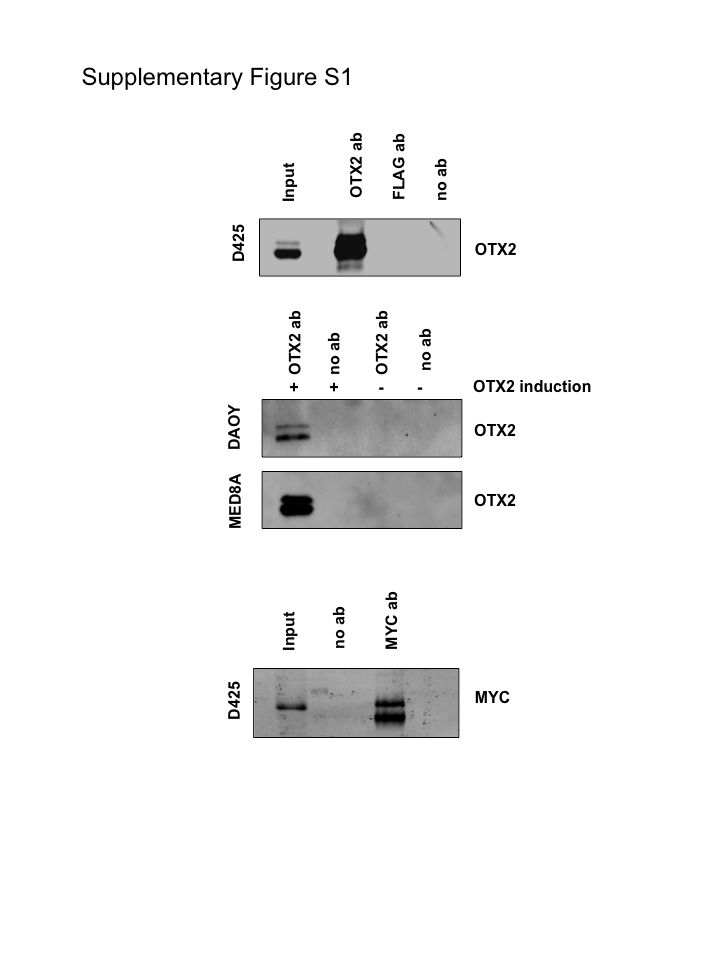

Supplement: Figure S1 — Immunoprecipitation of OTX2 and MYC in medulloblastoma cells. Western blot analyses of immunoprecipitation of OTX2 and MYC in D425 medulloblastoma cells as well as OTX2 in MED8A and DAOY cells with induced OTX2 expression. (TIFF) [file pone.0026058.s001.tiff]

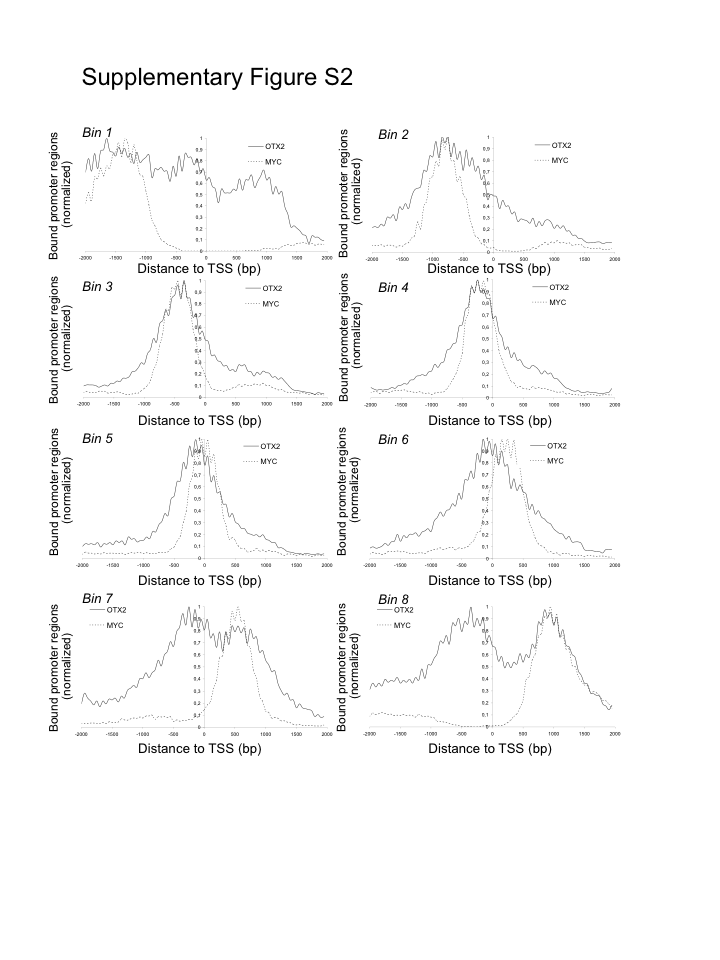

Supplement: Figure S2 — OTX2-binding distribution in relation to MYC binding. All promoter regions of the class with single OTX2-binding peak and MYC binding were sorted by the location of the first upstream MYC-binding peak. To assess the relation between MYC and OTX2 binding, the promoter regions were binned per 400 promoter regions, except for the last bin (517). Per bin, the average OTX2- and MYC-binding signals were calculated in regards to the TSS, normalized to 1 and depicted as a panel. The OTX2-binding signal coincides with the MYC, when the MYC binding signal becomes more proximal to the TSS. (TIFF) [file pone.0026058.s002.tiff]
